# Using impact monitoring mouthguards to measure head impact exposure in elite ice hockey

**DOI:** 10.1016/j.jsampl.2024.100069

**Published:** 2024-07-02

**Authors:** Mikael Swarén, Madelen Fahlstedt

**Affiliations:** a Swedish Unit for Metrology in Sports, School of Health and Welfare, Dalarna University, Falun, Sweden; b Neuronic Engineering, School of Chemistry, Biotechnology and Health, KTH Royal Institute of Technology, Stockholm, Sweden

**Keywords:** Concussion, Head injury, Injury prevention

## Abstract

**Background:**

Even though women's ice hockey does not permit deliberate checking between players, female players are at similar or even higher risk to sustain concussions, as male players. Several studies have investigated head impacts in ice hockey, however to the best of the authors' knowledge, no previous study has used impact monitoring mouthguards to investigate head impact exposure among professional female ice hockey players.

**Methods:**

Impact monitoring mouthguards were used to collect head impact data during games in the Swedish Women's Hockey League and in the men's Swedish J20 SuperElite League in 2020.

**Results:**

Female players had significantly higher median linear accelerations than male players (26 [19–35] g, vs. 7 [5–9] g, *p* ​< ​0.001, *d* ​= ​1.98). Female players had significant higher median rotational accelerations compared to male players (3076 [2314–4243] rad/s^2^ vs. 430 [281–752] rad/s^2^, *p ​<* ​0.001, *d* ​= ​2.398). There were no notable variances in impact distribution by location for linear or rotational accelerations among female players. Similarly, male players didn't exhibit significant differences in impact location for linear acceleration. However, impacts at the Top Front location demonstrated significantly higher rotational accelerations compared to those at Front Low and Front High positions.

**Conclusion:**

Compared to male players, female players sustain fewer but harder impacts to the head, which may explain the high occurrence of concussion in women's ice hockey.

## 1 Introduction

Ice hockey is a high-speed collision sport where players are exposed to several contact-related injury mechanisms, e.g., body checks, boards and ice contacts. Given the nature of the sport, ice hockey has a high injury rate, ranging from 5.95 to 9.5 per 1000 athlete exposures (AEs) and 5.12–6.1 per 1000 AEs, for male and female athletes, respectively [1,2]. Furthermore, ice hockey has a high concussion incidence, where concussions account for up to 14% of all hockey related injuries and up to 30% of all hockey related head injuries [[3], [4], [5]]. However, as reported by other studies [6,7], the concussion incidence is likely higher, due to the high numbers of concussions that are not reported, especially among female athletes. In contrary to men's ice hockey, women's ice hockey does not permit deliberate contacts (checking) between players. Still, previous research has shown that female ice hockey players are at similar or even higher risk to sustain concussions to males [1,2,[8], [9], [10], [11]]. The reason for the high risk among females is uncertain but may be related to anatomic and biomechanical differences e.g., head mass and neck strength [8,[12], [13], [14]]. Furthermore, La Fountaine el al [15]. found evidence that females could be more vulnerable to sustain a concussion during the late luteal phase of the menstrual cycle and the first few days of menstruation. Still, further studies are needed to fully understand the mechanisms behind the high occurrence of concussions among female athletes.

In ice hockey, several studies have investigated head impacts and concussions as well as exposure differences between male and female players. For instance, Mihalik et al. [16] investigated head impact exposure among youth ice hockey players (girls and boys, age 13–16 years) and showed that girls sustained fewer impacts per player compared to boys, but with higher linear accelerations. No significant difference between boys and girls regarding rotational accelerations was observed. These findings are in line with results by Eckner et al. [17] who compared head impact exposure between male and female high school ice hockey players and found that male players experience more head impacts compared to females whereas the mean impact magnitudes were greater for the female players. Furthermore, female players experienced more impacts to the side and top of the head compared the male players who had more impacts to the front and back [17].

Previous research [[18], [19], [20], [21]] has shown that material properties and helmet design can affect the impact absorption properties of ice hockey helmets. Female players must use a full-face cage whereas male players can choose to use a half-face visor from the age of 18. Benson et al. [22] reported a reduced concussion severity for ice hockey players who used a full-face cage compared to players using a half-face visor. Lemair & Pearsal [23] showed that a full-face cage reduce the peak linear accelerations of the head during impacts. In ice hockey, it is hence important to consider which type of helmets the players are using when comparing the magnitude of head impacts between different age groups, skill levels and gender.

Many studies investigating head impacts in sports have used the head impact telemetry (HIT) system (Simbex LLC, Lebanon, NH, USA) or other comparable helmed mounted systems consisting of a similar 6-accelerometer setup, fitted inside a helmet and transmitting data via radio frequency to a sideline receiver [16,[24], [25], [26], [27]]. However, several studies [[28], [29], [30], [31], [32], [33], [34]] suggest that data from helmet mounted systems need careful interpretation as the output data depend on sensor location, helmet design, helmet size and fit as well as the impact direction. There are other possible methods to analyse head impacts e.g., video analysis, sensor patches behind the ears and impact monitoring mouthguards (IMM). Higgins et al. [33] showed that the use of an IMM is more accurate to measure head accelerations, compared to a helmet mounted system as the IMM allow a direct assessment of the acceleration of the head and not the acceleration of the helmet. Liu et al. [35] compared and validated five of the most commonly used IMMs with a sensor equipped Hybrid III headform and found that all tested IMMs gave accurate measurements for the peak linear and rotational accelerations. Kieffer et al. [36] showed that accuracy of head impact sensors can vary between laboratory and field tests. They evaluated IMMs, helmet and skin mounted sensors and found that IMMs had the highest accuracy in both settings, even though some IMMs were less accurate in the field. However, previous research has shown that IMMs provide accurate impact data for everyday monitoring and can be used without video assessment of false positives/negative impacts [37,38].

Even though several studies have investigated head impacts in ice hockey has, to the best of the authors' knowledge, no previous study used IMMs to investigate head impact exposure among professional female ice hockey players. Hence, the main purpose of the present study was to investigate head impact exposure, location, and magnitude among professional ice hockey players in the Swedish Womens’ Hockey League (SDHL). A further purpose was to compare the data from the SDHL team with a male team in the Swedish J20 Super Elite league. Based on the high number of concussions among female ice hockey players, it was hypothesised that female and male elite ice hockey players are exposed to head impacts with similar magnitude and incident frequency.

## 2 Method

A total of 16 female ice hockey players (aged 20 ​± ​4 years) were recruited from a team, playing in the highest league in Sweden Swedish Women's Hockey League (SDHL). In addition, 20 male players (aged 19 ​± ​1 years) where recruited from a team playing in the J20 Super Elite league, which is the highest league in Sweden for players 19–20 years of age. All data were collected during 2020. The Swedish ethical board preapproved the study and experimental protocol (#2019–03310) and the study was conducted in accordance with the Declaration of Helsinki. All participants where fully informed of the nature of the study through written and verbal information before consenting to participate.

**Fig. 1:**
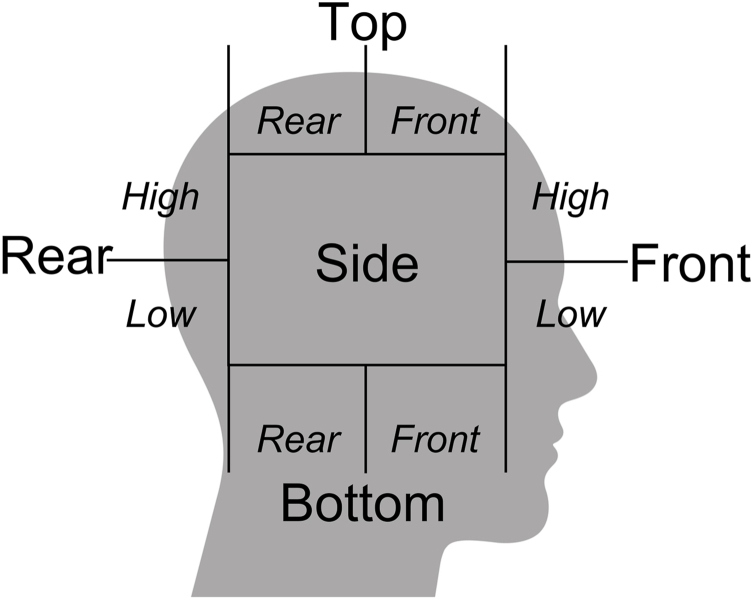
Pre-defined areas of impact location.

All participating players were equipped with an individually fitted “boil and bite” IMM (Prevent Biometrics, Edina, MN, USA) sampling linear and rotational velocity data at 3200 ​Hz, with a measurement range of ±200 ​g and ±35 ​rad/s [35]. Angular velocity is derived to calculate angular acceleration. The impact trigger threshold requires a single raw sample >5g on any single axis of the accelerometer data, including dynamic head movements caused by e.g., body checks. The 5g impact threshold was used in order to include indirect head acceleration events, as suggested by Tooby et al. [39]. The linear and rotational acceleration data from the IMMs were filtered at 200 ​Hz, 100 ​Hz and 50 ​Hz in order to properly report peak kinematics when real-world noises are present in the data from the playing field. The choice of filter was chosen to minimize the noise and maximize the head impact signal. While the exact filtering algorithms are proprietary, the assessment of waveform quality relies upon published data of head impact signal in the range of 20–80Hz and noise in the range of >200Hz from both laboratory and on-field data collection [40]. Acceleration data were automatically transformed to the estimated cranial center of mass by the Prevent Biometrics' software, with an output data time window of 50 ​ms. The Prevent Biometrics’ software also estimated the impact location based on pre-defined areas, Fig. 1.

Each IMM was fitted according to the product recommendation and all players were instructed to use the IMM for all planned games and practices during a period of three months.

Out of the 16 female players and 20 male players who agreed to participate in the study, eleven female players and eight male players used the IMMs continuously during all games. The female players played in lines 1–3, and the male players played in the first or second line. An ice hockey line consists of three forwards (including one center) and two defenders, where the first line normally is considered to consist of the most skilled players, followed by the second line, and so on. These lines rotate on the ice to keep the game intensity up. Lines one and two normally get the most play time during a game. Data were collected during eight SDHL games and six J20 Super Elite games as the leagues had to close due to the COVID-19 pandemic. All recorded impacts from practices were excluded as the players did not use the IMMs consequently during practices, making it difficult to analyse head impact exposure during practices. Also, not all practices could be video recorded and thus not allowing false positive impacts to be excluded. Hence, only head impacts from games are included in the results.

All recorded impacts were verified by video to eliminate any false positive impacts. To identify any false negative and positive head impacts, videos from all games were analysed by one experienced video analyst and one world class ice hockey official, as well as the head coaches for the two participating teams.

Due to the skewed nature of the data (Shapiro–Wilk test, p ​< ​0.05), all results are presented as median values and [25–75% interquartile range]. A two-way ANOVA with a Holm-Sidak post-hoc test for all pairwise comparisons, was used to examine the significance of the gender and location difference in impact magnitudes. A two-tailed p value of <0.05 was considered as statistically significant. All statistical analyses were performed using *jamovi* [41].

## 3 Results

A total of 38 and 68 head impacts were recorded during games for female and male players, respectively. During the period of the study, the head impact rate for female players was 0.4 impacts per game and 1.8 impacts per game for male players, resulting in a game exposure of 432 and 1833 head impacts per 1000 game exposure (GE) for female and male players, respectively.

**Fig. 2:**
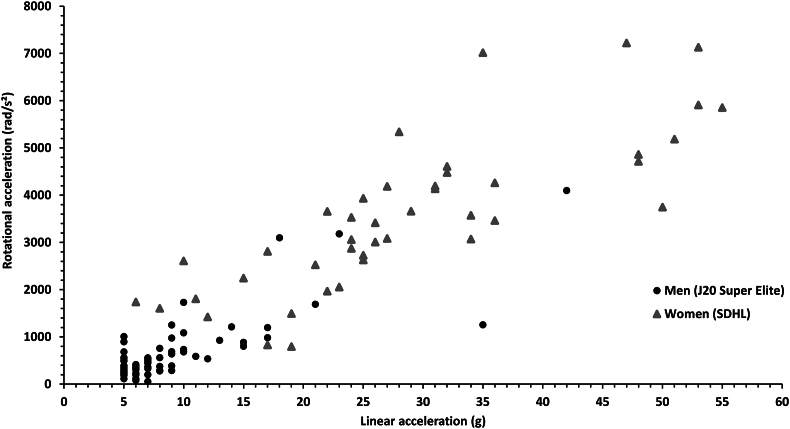
Linear and rotational accelerations for all head impacts.

**Fig. 3:**
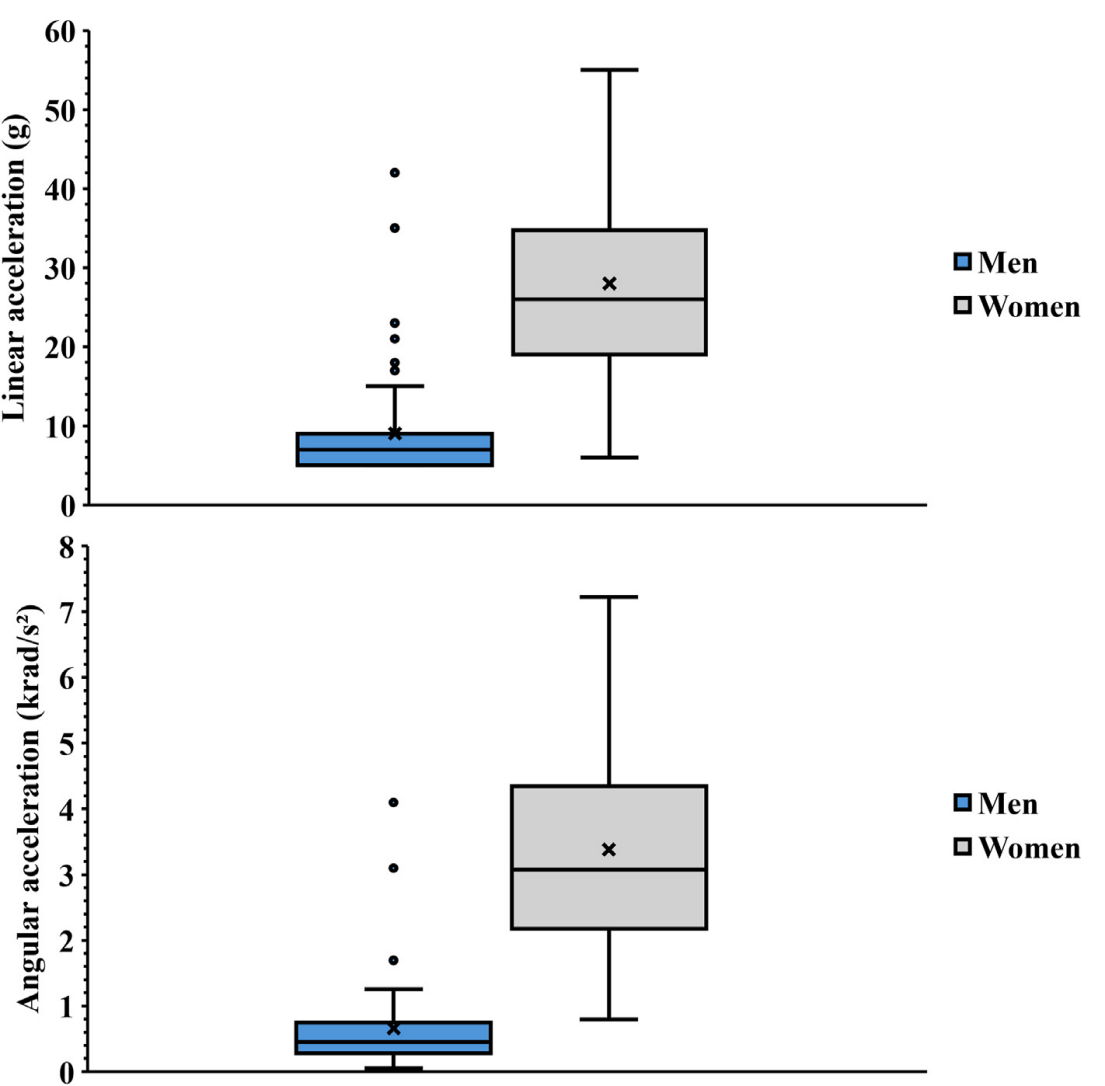
Distribution of linear and rotational accelerations for men and women respectively.

**Fig. 4:**
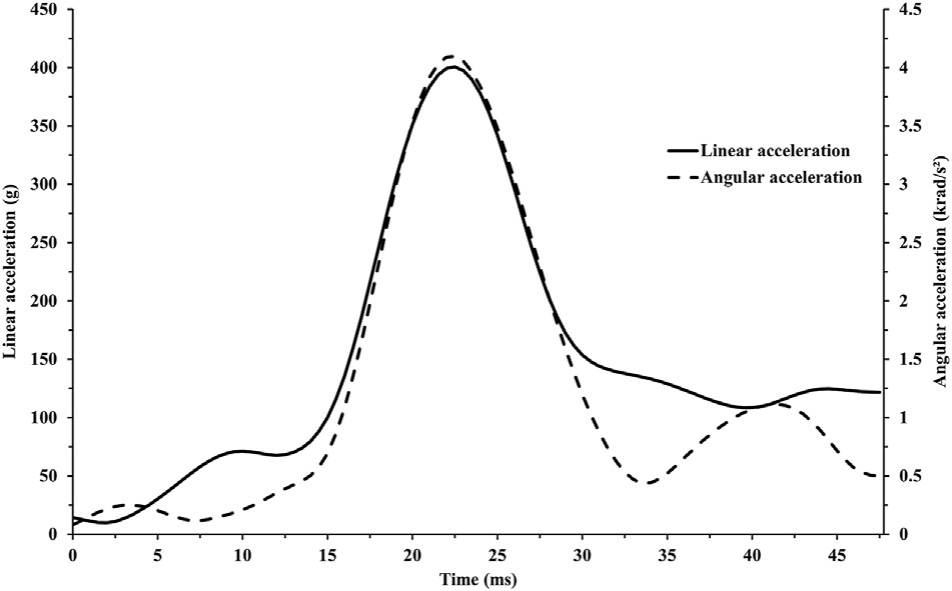
An example of the processed acceleration data for one head impact.

Female players were found to have significantly higher median linear accelerations than male players (26 [[19], [20], [21], [22], [23], [24], [25], [26], [27], [28], [29], [30], [31], [32], [33], [34], [35]] g vs. 7 [[5], [6], [7], [8], [9]] g, *p* ​< ​0.001, *d* ​= ​1.98). Female players were also found to have significant higher median rotational accelerations compared to male players (3076 [2314–4243] rad/s^2^ vs. 430 [281–752] rad/s^2^, *p ​<* ​0.001, *d* ​= ​2.398). The linear and rotational accelerations for all impacts are presented in Fig. 2, Fig. 3. An example of the processed acceleration data for one head impact can be seen in Fig. 4. None of the recorded impacts resulted in a diagnosed concussion.

Female players had no significant differences in distributions of impact magnitude by location in impact location for linear or rotational accelerations. Male players had no significant differences in impact in distributions of impact magnitude by location for linear accelerations, but significant differences were identified for rotational accelerations between Top Front and Front Low (895 [885–3182] rad/s^2^ vs. 320 [264–622] rad/s^2^, *p* ​< ​0.05, *d* ​= ​2.06) and Top Front and Front High (895 [885–3182] rad/s^2^ vs. 394 [258–518] rad/s^2^, *p* = ​< ​0.05, *d* ​= ​2.1). The locations of all impacts are presented in Table 1, and the individual head impact characteristics are presented in Table 2.

**Table 1:** Location of head impacts.

	Women (SDHL)	Men (J20 Super Elite)
Bottom front	3%	5%
Bottom rear	16%	9%
Front high	21%	14%
Front low	5%	17%
Left high	5%	8%
Left low	3%	18%
Rear high	0%	6%
Rear low	26%	2%
Right high	5%	6%
Right low	0%	5%
Top front	13%	8%
Top rear	3%	5%

**Table 2:** Head impact characteristics per player.

Female Players					
Player	Number of head impacts	Maximal PLA (g)	Mean PLA (g)	Maximal PAA (rad/s^2^)	Mean PAA (rad/s^2^)
W1	10	53	27 ​± ​16	5910	3223 ​± ​1366
W2	3	48	34 ​± ​13	4715	4325 ​± ​584
W3	2	36	27 ​± ​13	4259	3535 ​± ​1025
W4	4	34	22 ​± ​8	3070	1813 ​± ​1813
W5	1	28	–	5341	–
W6	4	47	27 ​± ​17	7223	4571 ​± ​2889
W7	2	31	28 ​± ​4	4193	3411 ​± ​1106
W8	1	19	–	792	–
W9	1	22	–	1966	–
W10	2	25	18 ​± ​11	2726	2667 ​± ​84
W11	8	55	35 ​± ​35	5855	3808 ​± ​1451
***Male Players***					
**Player**	**Number of head impacts**	**Maximal PLA (g)**	**Mean PLA (g)**	**Maximal PAA (rad/s**^**2**^**)**	**Mean PAA (rad/s**^**2**^**)**
M1	2	7	7 ​± ​1	466	290 ​± ​249
M2	14	23	9 ​± ​5	3182	788 ​± ​788
M3	13	21	9 ​± ​6	2099	887 ​± ​814
M4	12	15	7 ​± ​3	1732	520 ​± ​417
M5	17	42	9 ​± ​9	4097	664 ​± ​895
M6	8	35	12 ​± ​10	1255	469 ​± ​433
M7	1	7	–	535	–
M8	1	7	–	458	–

PLA ​= ​peak linear acceleration, PAA ​= ​peak angular acceleration. Mean values are presented as mean ​± ​SD.

Two false positives were detected during the video analysis and hence removed from the data set. No false negatives could be identified. The total number of triggered events is unknown due to proprietary algorithms.

## 4 Discussion

This is the first study where impact monitoring mouthguards (IMM) have been used in professional ice hockey, to monitor head impact exposure during games. As hypothesised, IMMs is a functional method to collect head impact data and to monitor head impact exposure among elite ice hockey players. However, contrary to our hypothesis, male and female players were not exposed to similar types of head impacts. Compared to male players, female players suffer fewer head impacts but with higher magnitude, which confirms previous findings by Eckner et al. [17]. Brainard et al. [42] also showed fewer impacts for female collegiate players, compared to male but that male players were more likely to sustain impacts with higher magnitudes. Contrary to Brainard et al. [42], head impacts among female players in the present study, showed higher median linear and rotational accelerations compared to male players. The median linear and rotational accelerations for female players are in line with results from reconstructed elbow and shoulder checks by Post et al. [43], who analysed head impacts in men's elite North American ice hockey, as well as with results from collegiate ice hockey by Wilcox et al. [44]. However, the present results are higher compared to previous results by Eckner et al. [17], who reported 18.8 ​g and 2778.2 ​rad/s^2^, as mean linear and rotational accelerations among high school female players, using impact-sensing adhesive skin patches to measure impact magnitudes and locations. Linear, and rotational accelerations for male players are lower in the present study, compared to previous reports [17,43,44]. Interestingly, Post et al. [43] report confirmed concussions at 30 ​g and 3500 ​rad/s^2^ impacts, received during reconstructed elbow and shoulder checks and Wilcox et al. [45] investigated nine concussion among female collegiate ice hockey players where the average linear and rotational accelerations were 43 ​± ​12 ​g and 4030 ​± ​1435 ​rad/s^2^, respectively. This suggests that several of the head impacts in the present study were high enough to potentially cause concussions, even though no concussion occurred during the data collection. However, the results by Post et al. [43] and Wilcox et al. [45] are lower compared to other reported impact levels for concussion. In e.g., American football, average concussions levels ranging between 61 and 169 ​g and 4235–9515 ​rad/s^2^ have been reported [[46], [47], [48], [49]]. Compared to linear kinematics, rotational kinematics have been suggested to be more strongly associated with concussions [[50], [51], [52], [53]]. However, it should be mentioned that the large variation of head impact levels between different studies is most likely explained by the use of helmed mounted impact sensors, which measure the impact of the helmet and not the head, and hence provide data with low accuracy and validity [[28], [29], [30], [31], [32], [33]]. Nevertheless, concussion thresholds levels are still unclear and further research is needed to increase the understanding of the different mechanisms, causing sports related concussion. Still, the median impact level among female players in the present study should be considered as high, with the potential to cause concussions.

As seen in Table 1, the majority of head impacts for female players were located in the front (42%) and rear (45%) of the head, whereas male players experienced more impacts on the sides (37%) and fewer rear impacts (21%), compared to female players. This is in contrary to findings by Eckner et al. [17] but similar with the results presented by Mihalik et al. [16]. However, both these studies investigate youth and high school players, whereas the present study included older players (>18 years), playing elite or professional ice hockey. The side impacts among male players have low magnitude and are received along the board and Plexiglas as male players are allowed more body contact and also to body check along the board and Plexiglas, contrary to female players. Still, male players have the same percentage of front impacts as female players but with more impacts around the face and chin areas, whereas front impacts for female players are located higher up on the head, which is in line with results by Brainard et al. [42]. This is likely because women's ice hockey does not allow deliberate checking between players, allowing female players to have a larger focus on the puck and hence a bent neck due to looking down. However, the present study does not analyse the different checking or collision situations causing the registered head impacts, as most of the situations for the female players were open situations, without a clear start or end of the movement/situation. Even though the actual impact events of the head were identified, e.g., when the head hit the ice or the sidewall, the events and actions causing the collisions and falls could not be categorized in a structured manner, as almost each situation was unique and could not be compared with the clear checking situations observed for the male players. However, future studies should categorize and analyse the situations leading to a head impact.

Even though the rulebook says that adult (>18 years) male players who play without a full-face cage must use a mouthguard during games, it is common that the majority of players in a team do not actually wear a mouthguard on the ice. Female players never use mouthguards as they must play with full-face cages. Hence, several players who originally signed up to participate in the study were not accustomed to use mouthguards and therefore only used the IMMs sparsely or not at all, as they felt disturbed when wearing the IMM. The data from these players are not included in the results as their usage of the IMMs do not represent a complete game-exposure. Hence, it is fair to assume that several head impacts went undetected due to this, lowering the total game-exposure value. However, the presented results for the players who used the IMMs consistently during the games are representative for these individual players. Still, future studies should ensure that all players in different teams always use the IMMs to investigate possible differences between the training and games for both male and female players, as well as getting valid athlete-exposure data throughout a complete season.

Due to the COVID-19 pandemic during the 2020 season, both leagues were stopped which limited the number of games for data collection and hence reduced the robustness of the statistical results. Still, eight female SDHL games and six J20 Super Elite games were recorded and a total of 104 head impacts were registered, resulting in a game exposure of 432 and 1888 head impacts per 1000 game exposure for female and male players, respectively. The higher exposure of head impacts for male players compared to female players is in line with what previous studies [17,54]. However, both Eckner et al. [17] and Wilcox et al. [54] report much higher head impact exposure per game compared to present study (7.7 and 6.3 impacts per game for men and 5.3 and 3.7 impacts per game for females). This is likely because they used a patch sensor behind the ear and a helmet mounted system, instead of an IMM system. This difference in head impact exposure between studies is mentioned as a major concern by Eckner et al. [17]. In other sports, head impact exposure per game are similar, compared to the present study. In women's collegiate rugby, Langevin et al. [55] report a head impact exposure of 0.4 impacts per game, with a threshold of 15 ​g. Interestingly, among high school soccer players, females have a higher head impact exposure per game, compared to male players (1.57 vs. 1.30 impacts per game) [56].

The higher head impact exposure for male ice hockey players is most likely explained by the different rules between men's and women's ice hockey where females are not allowed to body check. The present study used 5g as a cutoff level which allows a larger amount of low magnitude impacts to be recorded. King et al. [57] reported 10g to be the most commonly used threshold and recommended future studies to use a 10g threshold until more in-filed data are available. However, in a more recent study by Tooby et al. [39], both 5g and 10g thresholds were investigated, using rugby players in the field. They found that a 5g threshold is better for field tests as more indirect head acceleration events from e.g., tackles, are included in the data set. As checking is an essential part of ice hockey, a 5g threshold was used in the present study, to include indirect head acceleration events, and not just direct head impacts.

As mentioned by Eckner et al. [17], it is difficult to compare collected head impact data from the field as the results may differ from values recorded by other impact-sensing systems. Care should be taken when interpreting data from commercial systems as the exact filtering process is unknown due to proprietary algorithms. However, the IMMs used in the present study have been used and validated in several studies [35,36,40,58]. Hence, it is realistic to assume that the collected data are accurate and valid, and that future research should use IMMs to investigate head impact exposure in elite ice hockey. However, as mentioned previously, results from different leagues, age groups and gender need to be interpreted carefully as head impact data can be affected by helmet design and whether players are using full-cage or half-visor helmets. In the present study, the comparison between SDHL and J20 was considered the best option as SDHL is a professional league with older players, and likely more skilled players compared to e.g. 16- or 18-years old boys. Whereas the J20 players are very skilled but not as strong and physically developed as 25 years old professional players in the Swedish Hockey League (SHL). Also, most J20 players still play with a full face-cage which was important when evaluation the position of impacts etc. Still, it is important that future studies investigate impact exposure between professional male and female players as well as between different age groups.

## 5 Conclusions

In the present study, head impact monitors were used for the first time in women's professional ice hockey. Compared to male players in the Swedish J20 Super Elite league, female elite players suffer fewer but harder head impacts, with significant higher linear and rotational accelerations. Female players are most often hit in the front, or the rear of the head and male players are most often hit in the front, or on the sides of the head. Even though women's ice hockey does not permit deliberate checking between players, the present results show that female professional ice hockey players continuously are exposed to head impacts with the potential of causing concussion. It is therefore important to continuously monitor ice hockey players head impact exposure.

## Ethical statement

This research is not part of a larger study, and this manuscript represents results of original work that have not been published elsewhere. This manuscript has not and will not be submitted for publication elsewhere until a decision is made regarding its acceptability for publication in the Journal of Science and Medicine in Sport Plus. If accepted for publication, it will not be published elsewhere.

The Swedish ethical board preapproved the study and experimental protocol (#2019–03310) and the study was conducted in accordance with the Declaration of Helsinki. All participants where fully informed of the nature of the study through written and verbal information before consenting to participate. All authors acknowledge ethical responsibility for the content of the manuscript and will accept the consequences of any ethical violation.

## Funding details

This work was supported by the Nollvisionsprojektet, run by Swedish Ice Hockey.

## Declaration of competing interest

The authors declare no conflict of interest.
